# Multi-transcriptomics analysis of ferroptosis related genes reveals CAFs exosomal COX4I2 as a novel therapeutic target in osteosarcoma

**DOI:** 10.3389/fcell.2025.1620648

**Published:** 2025-09-04

**Authors:** Xiaoying Niu, Xinxin Zhang, Zhongyi Li, Wen Tian

**Affiliations:** ^1^ Bone and Soft Tissue Department, The Affiliated Cancer Hospital of Zhengzhou University and Henan Cancer Hospital, Zhengzhou, China; ^2^ The Second Clinical School, Nanjing Medical University, Nanjing, Jiangsu, China; ^3^ The Affiliated Cancer Hospital of Zhengzhou University and Henan Cancer Hospital, Zhengzhou, China; ^4^ The School of Public Health, Zhengzhou University, Zhengzhou, Henan, China

**Keywords:** osteosarcoma, prognostic signature, machine learning, ferroptosis, CAFs exosomal COX4I2

## Abstract

**Background:**

Osteosarcoma is a primary malignant tumor, characterized by its high incidence and recurrence rate in children and adolescents. Ferroptosis, an iron-dependent form of regulated cell death, has recently been recognized as a potential therapeutic vulnerability in cancer treatment. However, its prognostic significance and underlying regulatory mechanisms in osteosarcoma remain largely unexplored.

**Materials and methods:**

We constructed a prognostic model based on 12 ferroptosis-related genes using LASSO regression and validated across independent GEO cohorts (GSE21257 and GSE39055). We identified hub genes via machine learning algorithms (SVM, RF, XGBoost, BORUTA) and single-cell RNA sequencing. The exosomal transfer of COX4I2 protein from CAFs to 143B osteosarcoma cells was evaluated by Western blot, confocal microscopy, and transmission electron microscopy. Ferroptosis indicators, including Fe^2+^, MDA, ACSL4, and ROS levels, were assessed *in vitro*. We performed tumorigenicity assays *in vivo* in nude mice to validate biological function.

**Results:**

The ferroptosis-based risk model exhibited robust prognostic performance. We identified COX4I2 as a stromal hub gene, highly enriched in cancer-associated fibroblasts (CAFs). Functional experiments demonstrated that exosome-mediated delivery of COX4I2 suppressed ferroptosis in osteosarcoma cells and enhancd cell proliferation and mitochondrial integrity. Studies *in vivo* further revealed that overexpression of exosomal COX4I2 markedly promoted tumor growth while inhibiting ferroptosis.

**Conclusion:**

These findings underscore the potential of exosomal COX4I2 as a biomarker and therapeutic target for ferroptosis-based interventions in osteosarcoma.

## Graphical Abstract

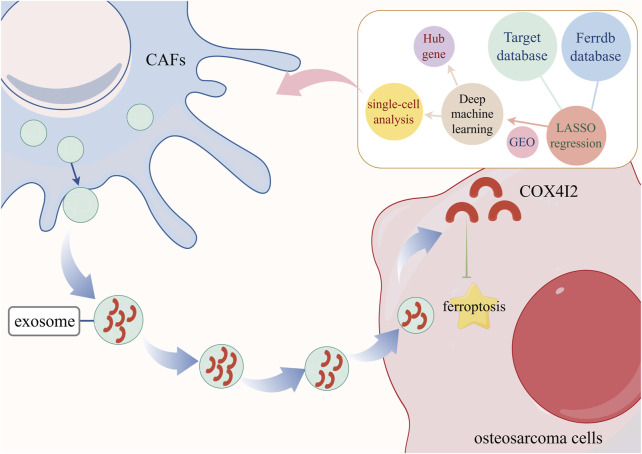

## Introduction

Osteosarcoma is the most prevalent primary malignant bone tumor in children and adolescents (Ritter and Bielack, 2010), marked by its high aggressiveness, early lung metastasis, and poor clinical prognosis (Kansara et al., 2014; Geller and Gorlick, 2010; Isakoff et al., 2015). The limitations of conventional therapies highlight the urgent need to explore alternative therapeutic vulnerabilities.

Increasing evidence underscores the critical role of the tumor microenvironment (TME) in disease progression (Belayneh et al., 2021). Among the diverse stromal components, cancer-associated fibroblasts (CAFs) constitute a predominant cell population that significantly modulates tumor behavior (Zhang et al., 2025; Pan et al., 2025). CAFs exert pro-tumorigenic effects via paracrine cytokines, extracellular matrix remodeling, and secretion of exosomes (Nedae et al., 2024). Exosomes derived from CAFs can transfer a broad spectrum of bioactive cargoes to regulate recipient cancer cell proliferation, resistance to apoptosis, immune evasion, and therapeutic tolerance (Peng et al., 2023). However, the specific exosomal proteins originating from CAFs and their functional significance in osteosarcoma progression remain incompletely understood.

COX4I2 (cytochrome c oxidase subunit 4 isoform 2) is a regulatory subunit of complex IV within the mitochondrial electron transport chain which modulate the efficiency of oxidative phosphorylation under varying oxygen concentrations (Pajuelo Reguera et al., 2020). In contrast to COX4I1, COX4I2 is selectively upregulated in low-oxygen environments and plays a role in metabolic adaptation and ROS regulation (Douiev et al., 2021). Emerging evidence have implicated COX4I2 in angiogenesis (Li et al., 2022) and tumor progression (Li JP. et al., 2024) in multiple malignancies. Nevertheless, its biological role in bone tumors, particularly in the context of exosome-mediated intercellular communication, remains largely undefined.

Ferroptosis is an iron-dependent form of programmed cell death characterized by excessive intracellular iron accumulation and lipid peroxidation (Torti and Torti, 2013; Xie et al., 2016; Dixon and Stockwell, 2014). Unlike apoptosis and necrosis, ferroptosis exhibits distinct morphological and biochemical features, such as mitochondria shrinkage with reduced cristae, increased mitochondrial membrane density, and elevated ROS production (Dixon and Olzmann, 2024; Chitambar, 2005). Several studies have established ferroptosis-related gene signatures as prognostic tools across various cancers. For instance, Elzbieta Panczyszyn et al. identified that inhibition of FSP1 activity could restore the sensitivity of osteosarcoma cells to ferroptosis (Panczyszyn et al., 2024). In recent years, ferroptosis has garnered increasing attention in oncology due to its potential influence on tumor malignancy (Minami et al., 2023; Zhou et al., 2024; Tang et al., 2020). However, there remains a paucity of systematic research focused on developing or validating ferroptosis-related prognostic signatures specifically for osteosarcoma.

Here, we initially developed a ferroptosis-related prognostic gene signature utilizing the TARGET cohort and validated its efficacy across multiple external GEO datasets. Through deep machine learning and single-cell RNA sequencing analysis, we identified COX4I2 as a stromal-enriched hub gene significantly associated with poor prognosis. Further investigations demonstrated that CAFs transfer COX4I2 to osteosarcoma cells through exosomes and suppressed ferroptosis by reducing intracellular Fe^2+^ levels and ROS accumulation, thereby promoting tumor proliferation. Collectively, these findings elucidated a novel regulatory axis involving CAFs, exosomal COX4I2, and ferroptosis, offering a promising therapeutic target for disrupting stromal support mechanisms in osteosarcoma.

## Materials and methods

### Data acquisition and processing

The TARGET database was used to acquire the ferroptosis-related genes expression data and corresponding clinical features of osteosarcoma.

The mRNA expression levels of GSE16091 and GSE21257 were extracted from the GEO database, utilizing the “limma” package for quality control and standardization. The GSE16091 dataset was based on the GPL96 platform, while the GSE21257 dataset utilized the GPL10295 platform. In total, there were 34 osteosarcoma tissue samples in the GSE16091 dataset and 53 osteosarcoma patients in the GSE21257 dataset. These two GEO datasets were employed as validation sets.

### Screening for ferroptosis-related genes

FerrDb online database was used to screen for ferroptosis-related genes (http://www.zhounan.org/ferrdb/current/operations/regulationnetwork.html). A total of 68 ferroptosis driver genes were collected.

### Establishing ferroptosis prognostic signature

The LASSO regression analysis was employed to construct prognostic features using the survival, survminer, and glmnet R packages (v4.1-2, 10-fold cross-validation). The ferroptosis prognostic signature was: riskscore= (0.2922) * ALOX15B+ (−0.248) * ATG7 + (−0.0004) * CD82 + (0.0768) * COX4I2 + (0.0426) * CYGB+(0.0103) * FADS2 + (−0.2002) *G6PD+(0.0283) * HILPDA + (−0.0797) * PPARG + (−0.3065) * SOCS1 + (−0.1363) * TRIM21 + (0.1109) * TRIM46.

The risk score was calculated based on gene expression levels and their corresponding coefficient values. Patients were stratified into high-risk and low-risk groups according to the risk scores, allowing for comparison of survival status and time between different risk groups. The predictive ability of the risk score in patient survival was evaluated using the ROC method. Furthermore, two independent CRC cohorts from the GSE16910 and GSE21257 databases were used to validate the prognostic values of ferroptosis signature. The immunological scores of high-risk and low-risk groups were analyzed based on clinical data. Calculation analysis was performed using the R immunedeconv package, and the results were visualized using the R ggplot2 software package.

### Machining learning to screen key ferroptosis-related genes

The 12 ferroptosis-related genes were screened using SVM, RF, XG-Boost, and the BORUTA algorithm. The SVM is a supervised learning classification method that aims to identify a hyperplane in the feature space, maximizing its distance from all other data points, and effectively separating different categories of data to ensure optimal performance of the constructed prognostic model (Elemam and Elshrkawey, 2022). The RF algorithm is employed as a regression algorithm for ensemble learning based on decision trees, enabling faster training processes and mitigating estimation bias (Zhang et al., 2023). The XG-Boost algorithm is an integrated learning method that leverages gradient boosting decision trees. It incorporates a regularization term and utilizes second-order derivative information to enhance model performance and generalization capabilities. By combining multiple weak decision trees, it forms a robust classifier. Each tree is trained based on the residual of the previous tree, with iterative optimization of the loss function gradually reducing the residual. Simultaneously, the model controls tree complexity and regularization terms to mitigate overfitting risks (Hu et al., 2023). The BORUTA algorithm introduces stochasticity into the system and subsequently obtains outcomes from a collection of random samples in order to mitigate the confounding effects of random fluctuations and correlations. This additional randomness provides a more discerning depiction of the variables that truly hold significance (Friedman, 2001).

### Pathway prediction

The ssGSEA algorithm was employed to calculate the correlation between gene expression and pathway scores, facilitating the elucidation of the interplay between genes and pathways.

### Single-cell analysis

Single-cell transcriptomic data from osteosarcoma samples were retrieved from the GEO database (GSE152048). The Seurat R package (v4.3.0) was employed for data preprocessing, dimensionality reduction, and clustering. Cells with fewer than 200 or more than 6000 detected genes, as well as those exhibiting greater than 10% mitochondrial gene content, were excluded from further analysis. The gene expression matrix was standardized using the NormalizeData function with the log-normalization method. Subsequently, 2000 highly variable genes were selected for further analysis. Batch effects were corrected employing the Harmony package (v0.1.0). Dimensionality reduction was carried out via principal component analysis (PCA), followed by clustering analysis using the FindClusters function at a resolution of 0.5. For two-dimensional visualization, the UMAP algorithm was applied. Cell types were annotated based on the expression profiles of canonical marker genes. Differentially expressed genes (DEGs) were identified using the FindMarkers function in combination with the Wilcoxon rank sum test.

### Cell culture

143B cells were cultured in a complete medium consisting of RPMI DMEM supplemented with 10% FBS and double antibody, at a temperature of 37 °C and under a CO_2_ concentration of 5% in the incubator.

### CAFs isolation and identification

Primary CAFs were isolated from freshly collected osteosarcoma tissue specimens obtained intraoperatively from patients who had provided informed consent. The tumor tissues were cut into approximately 1 mm^3^ fragments and subsequently digested with 0.2% type I collagenase (Sigma-Aldrich, C0130) and 0.1% hyaluronidase (Sigma-Aldrich, H3506) at 37 °C for 2 h under continuous agitation. Following digestion, the resulting cell suspension was filtered through a 70 μm cell strainer, centrifuged, and then resuspended in Dulbecco’s Modified Eagle Medium (DMEM) supplemented with 10% FBS and 1% penicillin/streptomycin. The cells were cultured at 37 °C in a humidified atmosphere containing 5% CO_2_, and fibroblast-like adherent cells were selectively enriched through sequential medium changes. The identity of the isolated CAFs was confirmed by morphological assessment, immunofluorescence staining for α-SMA, Vimentin, and E-cadherin. To further verify purity, flow cytometric analysis was conducted, and only CAFs exhibiting greater than 90% α-SMA positivity were used for subsequent experiments.

### Exosome isolation and characterization

Exosomes were isolated from conditioned medium through a sequential centrifugation protocol, including 300 × g for 10 min, 2,000 × g for 20 min, and 10,000 × g for 30 min, followed by ultracentrifugation at 100,000 × g for 70 min (Beckman Coulter Optima L-100K). Following ultracentrifugation, exosomes were resuspended in phosphate-buffered saline (PBS) and pelleted again under the same ultracentrifugation conditions. Particle size distribution and concentration were analyzed using nanoparticle tracking analysis (NTA, NanoSight NS300). Morphological characterization was performed using transmission electron microscopy (TEM, JEOL JEM-1230). The presence of exosomal surface markers (CD63, Alix, and TSG101) were confirmed via Western blot analysis.

### Western blot

The cells were lysed using RIPA (KGA5203-100, KeyGEN BioTECH) buffer to isolate the total protein. The protein concentration was determined using a BCA protein assay kit (KGA2101-250, KeyGEN BioTECH). Subsequently, the total protein was separated by SDS-PAGE and transferred onto a 0.45 μm nitrocellulose membrane. After blocking the membrane with skim milk powder at room temperature for 1 h, it was incubated overnight in a 4 °C shaker with the primary antibody. Monoclonal primary antibodies were used: CD63 (ab134045, Abcam), TSG101 (ab125011, Abcam), and Alix (ab275377, Abcam), COX4I2 protein (ab202554, Abcam), ACSL4 (ab155282, Abcam), and anti-β-actin (66009-1-Ig, Proteintech). Following overnight incubation, the membrane was combined with the corresponding goat anti-mouse secondary antibody (SA00001-1, Proteintech) was then incubated at room temperature for 1h. Finally, the protein bands were visualized using SuperSignal ECL kit (KGC4602-200, KeyGEN BioTECH) and imaged.

### qRT-PCR

Total RNA was extracted from 143B cells that had been co-cultured for 24 h with or without exosomes derived from cancer-associated fibroblasts (CAFs), using TRIzol reagent (Invitrogen). Complementary DNA (cDNA) was synthesized using the PrimeScript RT Reagent Kit (RR047A, Takara). Quantitative real-time PCR was conducted using TB Green Premix Ex Taq II (RR820A, Takara) on a StepOnePlus Real-Time PCR System (Applied Biosystems). The following primers were used:

COX4I2 forward: 5′-AGTGGTGGCTCTTCAGATCG-3′

COX4I2 reverse: 5′-CTGAGTTGATGGCAGGATGA-3′

GAPDH forward: 5′-GGTGAAGGTCGGTGTGAACG-3′

GAPDH reverse: 5′-CTCGCTCCTGGAAGATGGTG-3′

Relative expression was calculated using the 2^−ΔΔCt^ method. GAPDH served as the internal control. Three biological replicates were performed.

### GW4869 treatment

To suppress exosome secretion from CAFs, we employed the neutral sphingomyelinase inhibitor GW4869 (SML0100, Sigma-Aldrich, United States). CAFs were cultured to reach 60%–70% confluence in complete growth medium and subsequently exposed to 10 μM GW4869 for 24 h. Following the treatment period, the culture supernatant was harvested and processed for exosome isolation using the previously established protocol.

### siRNA transfection

CAFs were transfection with NC or COX4I2 protein overexpression plasmid constructs provided by RIBOBIO Biotechnology Company. Transfections were performed using Lipofectamine™ 3000 (L3000075, ThermoFisher) for delivery of NC or COX4I2 protein overexpression plasmid. All experiments were conducted 48 h post-transfection.

### Edu assay

The 143B cells were treated with Edu (50 μM in medium) and incubated in a 5% CO_2_ incubator at 37 °C for 2 h. Subsequently, the medium was discarded and the cells were washed twice with 1 × PBS. Afterwards, the cells were fixed by adding 500 μL of cell fixative (1 × PBS containing 4% paraformaldehyde) and incubating at room temperature for 30 min. The fixative was then removed, followed by addition of 200 μL of a glycine solution (2 mg/mL). After decolorization and a further incubation period of 5 min, the glycine solution was discarded. Next, the cells were washed with 1 × PBS using a decolorizing shaker for 5 min. Each sample was then treated with Apollo® dyeing solution (KGA9606-100, KeyGEN BioTECH) by adding to a volume of 500 μL. Incubation at room temperature in a decolorizing shaker for an additional duration of 30 min was performed before discarding the dyeing solution. To clean any remaining dye from the samples, they were subjected to multiple washes using penetrant (0.5% TritonX-100 PBS), each lasting for 10 min on a decolorizing shaker. A sufficient amount of Hoechst33342 reaction solution was prepared and added in an amount totaling 500 μL. The resulting solutions were then incubated at room temperature away from light on a decolorizing shaker for15min, followed by one-to three additional cleanings with PBS for fluorescence microscope imaging.

### Fe^2+^ assay

The intercellular iron levels were determined by iron colorimetric assay kit (E-BC-K881-M, Elabscience, China). The cells were chilled and subsequently centrifuged at 15,000 g. Then the supernatant was collected and reagents containing iron reductase were added. After a 40 min incubation, the optical density was measured using a multiscan spectrum at 593 nm.

### MDA detection

Lipid peroxidation was assessed by measuring MDA levels using a commercial MDA assay kit (S0131S, Beyotime, China), according to the manufacturer’s instructions. In brief, 143B cells were treated with PBS, control CAFs-derived exosomes, or COX4I2-overexpressing CAFs-derived exosomes for 24 h. Following treatment, cell lysates were mixed with thiobarbituric acid (TBA) reagent and incubated at 95 °C for 40 min. After cooling to room temperature, the samples were centrifuged at 10,000 × g for 10 min, and the absorbance of the resulting supernatant was measured at 532 nm using a microplate reader.

### ROS assay

The ROS levels measures were measured using corresponding commercial kits (KGA7308, KeyGene Biotech). The DCFH-DA loading working solution was prepared by diluting it at a ratio of 1:1000 to achieve concentration of 10 µM. The cells were prepared with 1 × 10^6^ cells/mL. After removing medium, the cells were washed twice with PBS and then incubated with the DCFH-DA working solution at 37 °C for 20 min. Subsequently, the DCFH-DA working solution was removed and the cells were washed three times with serum-free medium. This protocol enables fluorescence detection using a multi-functional enzyme marker.

### Observation and quantitative analysis of mitochondria

The ultrastructural characteristics of mitochondria in 143B cells under various treatment conditions were systematically evaluated using transmission electron microscopy (TEM). The mitochondrial area (μm^2^) and the number of mitochondria per cell were quantified using ImageJ software. For each experimental group, at least 10 cells were randomly selected, and more than 100 individual mitochondria were manually traced and measured to ensure accurate quantification.

### Animal assay

Four-week-old nude mice were obtained from Shanghai Lingchang Biotechnology Co., Ltd (China). These studies were approved by the Nanjing Lamu Pharmaceutical Co., LTD (No. 20220038).

Mice were randomly divided into two groups: (A) exosomal COX4I2 protein overexpression group (n = 3) and (B) NC group (n = 3). Each mouse was subcutaneously injected with 143B cells (1 × 10^7^). When the tumor volume reached approximately 100 mm^3^, the mice in groups A and B received specific treatments. Tumor formation was monitored and tumor size was calculated using formula V = 0.5 × L × W^2^, where L is the length and W is the width of the tumor.

At the conclusion of the assay, a high dosage of intravenous barbiturates was administered to the experimental mice, ultimately inducing a painless and unconscious state followed by cessation of respiration.

### Immunohistochemistry (IHC)

Tumor tissues from xenograft mice were fixed in 4% paraformaldehyde, embedded in paraffin, and sectioned at a thickness of 5 μm. The tissue sections were deparaffinized, rehydrated, and subjected to antigen retrieval using citrate buffer (pH 6.0). Endogenous peroxidase activity was blocked with 5% bovine serum albumin (BSA), followed by overnight incubation at 4 °C with a primary antibody against COX4I2 (ab202554, Abcam) and GPX4 (ab125066, Abcam). After thorough washing, the sections were incubated with an HRP-conjugated secondary antibody (ZSGB-BIO, China), developed using 3,3′-diaminobenzidine (DAB) substrate, and counterstained with hematoxylin. The stained sections were visualized and imaged using a Nikon Eclipse microscope.

### Statistical analysis

The analysis of the data was conducted using R studio software. Group comparisons were carried out using the student t test. The value of *P* < 0.05 was set as statistically significant differences.

## Results

### Construction of the ferroptosis predictive signature

**FIGURE 1:**
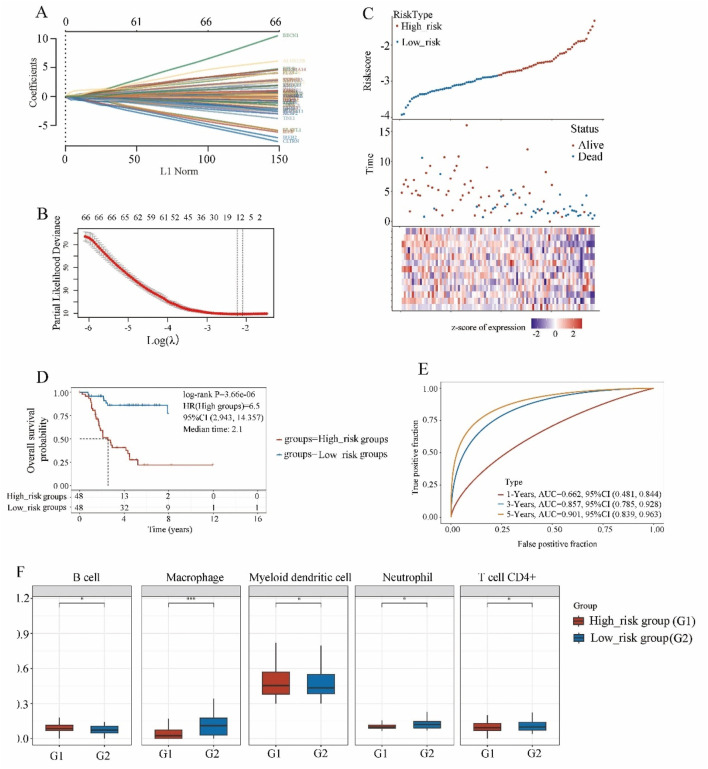
The screening of ferroptosis candidate prognostic genes using LASSO machine learning algorithms. **(A)** Coefficient RIF profile plot of the LASSO model showed the selection of the optimal parameter l (lambda). **(B)** Tenfold cross-validation to select the optimal tuning parameter log(lambda) in the TARGET osteosarcoma dataset (n = 96). **(C)** Risk survival status plot of ferroptosis signature in the TARGET osteosarcoma dataset. **(D)** Kaplan-Meier curves result of high-risk group (n = 48) and low-risk group (n = 48). **(E)** The AUC of the prediction of 1, 3, 5-year survival rate of osteosarcoma. **(F)** The immune scores of high-risk group and low-risk group.

The expression matrices of 96 osteosarcoma patients were obtained from the TARGET website. A total of 68 driver genes associated with ferroptosis were acquired from the FerrDb online database (Supplementary Table S1). Through LASSO regression analysis, we identified 12 hub genes, which included ALOX15B, ATG7, CD82, COX4I2, CYGB, FADS2, G6PD, HILPDA, PPARG, SOCS1, TRIM21, and TRIM46, and developed a prognostic model (Figures 1A,B). As depicted in Figure 1C, the risk score was evaluated, demonstrating that the ferroptosis model effectively discriminated between the high-risk group and the low-risk group. Based on this model, risk scores for osteosarcoma patients in the TARGET database were calculated and divided into high-risk and low-risk groups, each consisting of 48 patients.

The data was analyzed using the Kaplan-Meier curve was used to analyze the data (Figure 1D). Compared to the low-risk group, the high-risk group exhibited a shorter survival time and a poorer prognosis (HR = 6.5, *P* < 0.001). Furthermore, the ROC curve demonstrated that at 1, 3, and 5 years, the model had robust predictive ability with AUC values of 0.662, 0.857, and 0.901 respectively (Figure 1E, *P* < 0.000), indicating its efficacy in predicting osteosarcoma prognosis at 3 and 5 years.

Multivariate Cox regression analysis demonstrated that the risk score was significantly correlated with overall survival (*P* < 0.01), irrespective of age, gender, disease stage, and metastasis status (Supplementary Table S2), which highlighting the potential clinical application of the 12-gene ferroptosis-based model as an effective prognostic indicator.

The immune scores (Figure 1F) of the high-risk group and low-risk group exhibited significant correlations with B cells, macrophages, myeloid dendritic cell, neutrophil, and T cells CD4^+^.

### Independent validation of ferroptosis signature prognostic values

**FIGURE 2:**
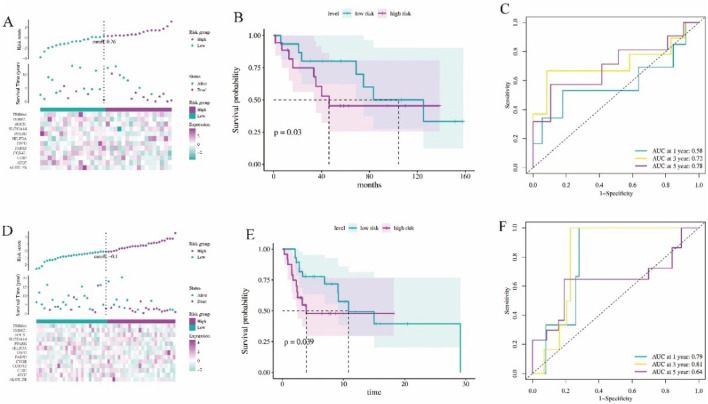
The validation of the ferroptosis signature diagnostic values. **(A)** Risk survival status plot of ferroptosis signature in GSE16091 dataset (n = 34). **(B)** Kaplan-Meier curves result of high-risk group and low-risk group in GSE16091 dataset. **(C)** The AUC of the prediction of 1, 3, 5-year survival rate of GSE16091 dataset. **(D)** Risk survival status plot of ferroptosis signature in GSE21257 dataset (n = 53). **(E)** Kaplan-Meier curves result of high-risk group and low-risk group in GSE21257 dataset. **(F)** The AUC of the prediction of 1, 3, 5-year survival rate of GSE21257 dataset.

To further assess the association between 12 ferroptosis-related gene signatures and patient survival, we acquired gene expression and clinical data from independent cohorts of patients in GSE16091 and GSE21257. Patients from GSE16091 were divided into high-risk and low-risk groups (Figure 2A). Kaplan-Meier analysis revealed that patients with high-risk scores exhibited significantly poorer overall survival compared to those with low-risk scores (Figure 2B, *P* = 0.03). Additionally, ROC analysis demonstrated that the ferroptosis signature displayed enhanced accuracy in predicting patient prognosis, as evidenced by AUC values exceeding 0.58, 0.72, and 0.78 for 1, 3, and 5-year predictions, respectively (Figure 2C). Similarly, osteosarcoma patients with GSE21257 analysis were effectively stratified into high-risk and low-risk groups (Figure 2D). Notably, patients in the high-risk group exhibited significantly shorter survival and poorer prognosis compared to those in the low-risk group (Figure 2E, *P* = 0.039). The results of ROC analysis demonstrated that the AUC was largest for 3-year prognosis, reaching 0.81 (Figure 2F), indicating a robust diagnostic efficiency. Collectively, these findings underscore the prognostic potential of the ferroptosis-related gene signature.

### Identification of hub ferroptosis-related genes through deep machine learning

**FIGURE 3:**
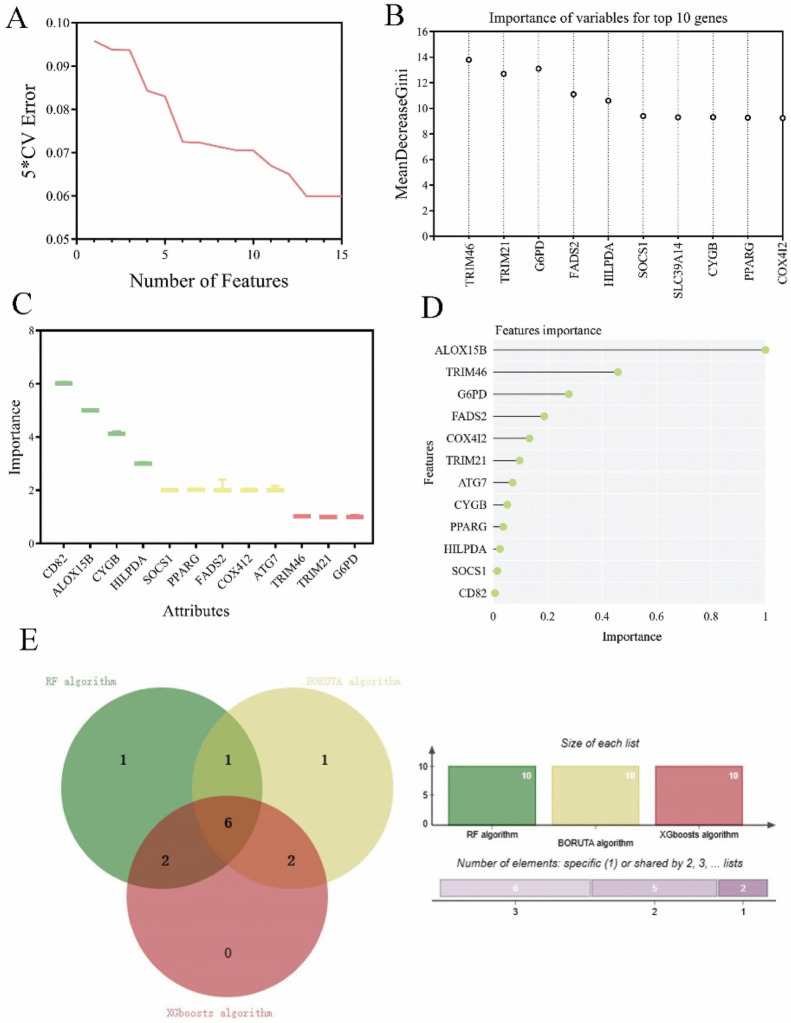
Exploration of ferroptosis signature genes using machine learning algorithms. **(A)** The results of SVM-RFE algorithm. **(B)** The top 10 genes of ferroptosis candidate prognostic genes according to their discriminant ability in the RF algorithm. **(C)** The selection of shared ferroptosis candidate prognostic genes by using the BORUTA algorithm. **(D)** The selection of twelve ferroptosis signature genes by using the XGBoosts algorithm. **(E)** The Venn map of common ferroptosis signature genes of RF algorithm, BORUTA algorithm, and XGBoosts algorithm.

Next, we performed deep machine learning algorithms to identify the hub gene from the ferroptosis-related gene signature. Firstly, the result of SVM algorithm showed the lowest 5 * CV error of 12 genes in this prognostic signature (Figure 3A). The RF machine learning algorithm was then employed to rank these 12 candidate genes based on their variable importance, and the top 10 significant genes were extracted and displayed with their MeanDecreaseGini scores in Figure 3B. Additionally, we utilized the R packages “Boruta” and “mlbench” to execute the BORUTA algorithm to calculate the 10 most significant genes (Figure 3C). Subsequently, the top 10 genes of these aforementioned 12 genes were inputted into an XGboosts algorithm classifier for final selection (Figure 3D).

According to the above three distinct machine algorithms, we have identified a cumulative of six most important genes: TRIM46, FADS2, HILPDA, CYGB, PPARG, and COX412 (Figure 3E).

**FIGURE 4:**
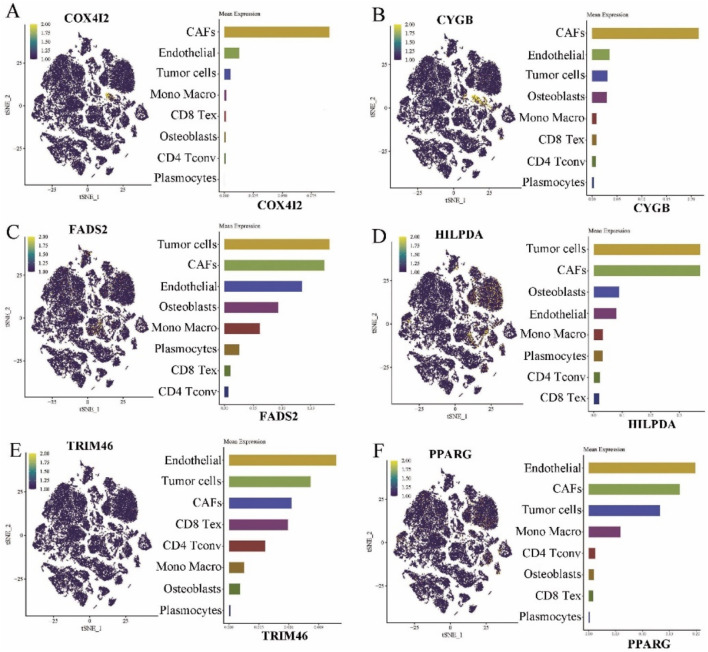
Results of single cell analysis of ferroptosis signature prognostic model genes in GSE162454 (n = 6). **(A)** COX4I2. **(B)** CYGB. **(C)** FADS2. **(D)**. HILPDA. **(E)** TRIM46. **(F)** PPARG.

Additionally, we conducted single-cell analysis on GSE162454 to investigate the distribution of the above 6 genes in various cellular components of osteosarcoma tissues. As illustrated in Figures 4A,B, COX4I2 and CYGB exhibited enrichment specifically in CAFs. FADS2 (Figure 4C) and HILPDA (Figure 4D) showed significantly elevated levels in tumor cells. And TRIM46 (Figure 4E) and PPARG (Figure 4F) demonstrated high expression levels in endothelial cells.

To further validate the cellular origin of COX4I2 within the tumor microenvironment, we examined its expression across annotated cell populations using single-cell RNA sequencing data derived from osteosarcoma samples. The overlay of COX4I2 expression onto the UMAP projection showed pronounced spatial enrichment specifically within the CAFs cluster (Supplementary Figure S8A). To quantify this observation, we compared COX4I2 expression levels across tumor cells and CAFs using violin plots. Notably, COX4I2 expression was significantly elevated in CAFs compared to tumor cells (Supplementary Figure S8B, *P* < 0.001). The significant contribution of the COX4I2 gene in the prognostic signature and its aberrant enrichment in CAFs prompted us to further investigated the molecular mechanism underlying the COX4I2 gene.

### COX4I2 was associated with poor prognosis of patients

**FIGURE 5:**
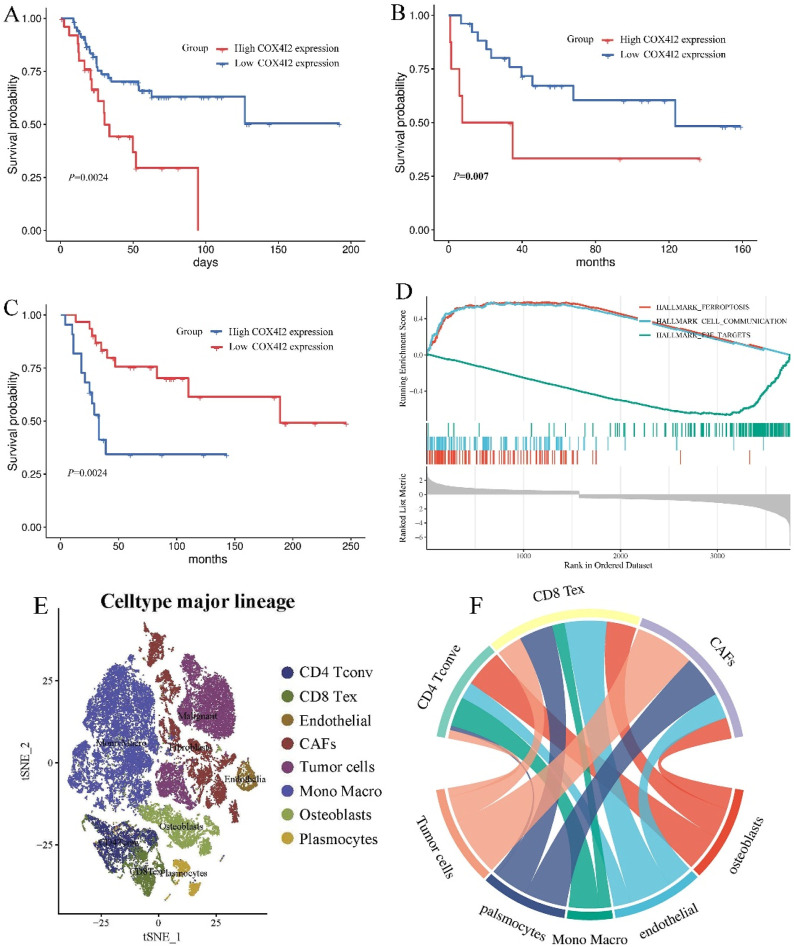
The association between COX4I2 expression levels and prognosis of patients with osteosarcoma. **(A)** The prognostic analysis of COX4I2 expression level in TARGET osteosarcoma dataset (high COX4I2 group: n = 48, low COX4I2 group: n = 48), **(B)** GSE16091 (high COX4I2 group: n = 15, low COX4I2 group: n = 19), and **(C)** GSE21257 (high COX4I2 group: n = 22, low COX4I2 group: n = 31). **(D)** GSEA result of COX4I2. **(E)** The celltype major lineage of GSE162454. **(F)** The chord diagram of cell-to-cell communication of GSE162454.

The Cox analysis results of TARGET database (Figure 5A), GSE16091 (Figure 5B), and GSE21257 (Figure 5C) revealed that patients with high COX4I2 expression exhibited significantly shorter survival time compared to those with low COX4I2 expression in osteosarcoma. This observation strongly suggested a significant association between aberrant COX4I2 expression and unfavorable prognosis in osteosarcoma patients. Furthermore, GSEA analysis revealed that abnormal COX4I2 expression was associated with intercellular communication, ferroptosis, and E2F targets (Figure 5D).

Single-cell RNA sequencing analysis of the GSE162454 dataset revealed robust cellular communication between CAFs and osteosarcoma tumor cells (Figures 5E,F). This suggested that CAFs may induce malignant characteristics in osteosarcoma by the interactation between tumor cells with osteosarcoma cells through the transmission of COX4I2 protein by mediating ferroposis.

To further elucidate CAFs - tumor communication, we performed ligand - receptor interaction analysis. This revealed several dominant signaling axes included IL6 – IL6R, GDF15 – TGFBR2T, and VEGFA–NRP1 (Supplementary Figure S10).

### CAFs transferred COX4I2 to 143B cells by exosomes

**FIGURE 6:**
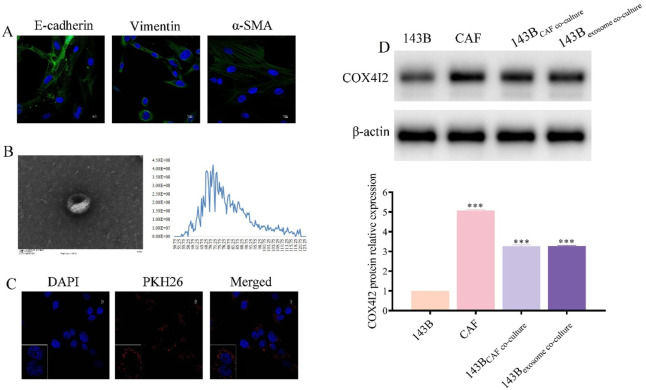
COX4I2 protein was transferred from CAFs to 143B via exosomes. **(A)** Characterization results of CAFs. **(B)** Characterization results of exosomes derived from CAFs. **(C)** Confocal microscopy results after co-culturing exosomes with 143B for 24 h. **(D)** Western blot results of COX4I2 protein expression bands in 143B, CAFs, and 143B in different co-culture groups. Statistical analysis: One-way ANOVA with Tukey’s *post hoc* test. n = 3 biological replicates. Data shown as mean ± SD. ***P < 0.001.

To validate the aforementioned hypotheses, we initially isolated CAFs from tumor tissues and performed cell characterization and identification. The results were presented in Figure 6A, where the expression of E-cadherin, Vimentin, and α-SMA were clearly detectable, confirming that the isolated cells were indeed CAFs and suitable for subsequent experiments.

Furthermore, we extracted exosomes derived from CAFs and conducted corresponding characterizations. As shown in Figure 6B, the vesicles exhibited complete cup-shaped structures with diameters predominantly ranging between 50 nm and 120 nm. To further confirm the exosomal identity, we conducted Western blot analysis using classical exosome-specific markers. As illustrated in Supplementary Figure S1, the presence of CD63, TSG101, and Alix was clearly detected, thereby verifying that the isolated extracellular vesicles were exosomes and suitable for downstream functional experiments.

Following a 24-h co-culture of CAFs-derived exosomes with 143B, we observed that 143B cells effectively phagocytosed the CAFs-derived exosomes (Figure 6C; Supplementary Figure S2). COX4I2 mRNA and protein were highly expressed in CAFs-derived exosomes, consistent with its expression levels in CAFs. Following co-cultivation of CAFs-derived exosomes with 143B cells, elevated expression of COX4I2 was also observed in 143B cells, suggesting that the COX4I2 mRNA and protein may be transferred from CAFs-derived exosomes. Furthermore, when exosome secretion from CAFs was inhibited using GW4869 and subsequently co-cultured with 143B cells, the expression of COX4I2 in 143B cells was significantly reduced (Figure 6D; Supplementary Figures S3, S4, *P* < 0.001). This indicated that the upregulation of COX4I2 in 143B cells was mediated by exosomal transfer from CAFs. This resulted in an increased expression of the COX4I2 protein in 143B cells.

### COX4I2 derived from CAFs exosomes inhibited ferroptosis in 143B *in vitro*

**FIGURE 7:**
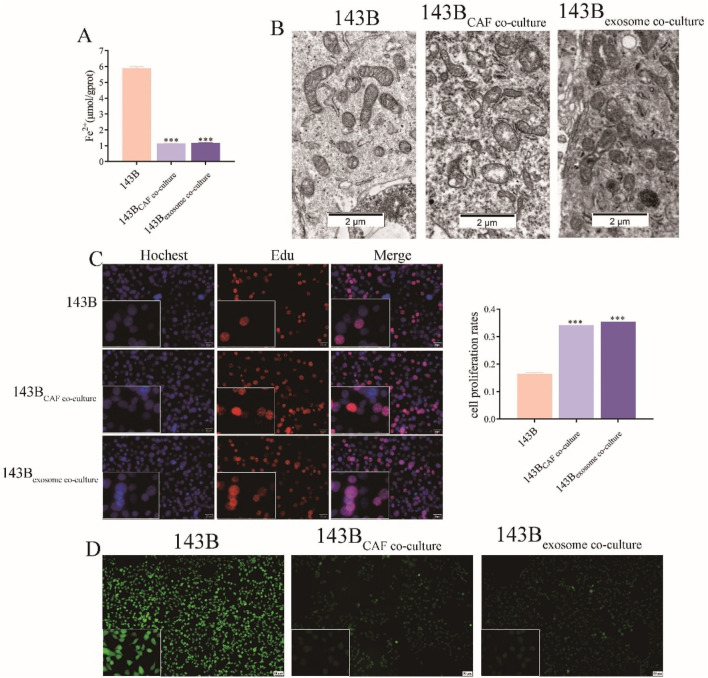
CAF-derived exosomal COX4I2 protein inhibited ferroptosis in 143B. **(A)** Fe^2+^ accumulation detection. **(B)** Observe the morphology of mitochondria by electron microscopy. **(C)** Edu cell proliferation results. **(D)** ROS fluorescence detection results. Statistical analysis: One-way ANOVA; n = 3 replicates. Data presented as mean ± SD. ***P < 0.001.

Combined with the prediction results presented in Figure 5, we conducted an analysis of ferroptosis in 143B following a 24-h co-culture. As illustrated in Figure 7A, compared to 143B cells, there were reductions in Fe^2+^ accumulation in 143B co-cultured with CAFs and CAFs derived exosomes. Similarly, the levels of MDA (Supplementary Figure S9A, *P* < 0.001) and ACSL4 (Supplementary Figure S10A, *P* < 0.001) were found to be lower in 143B cells co-cultured with CAFs as well as in exosomes derived from CAFs, when compared to the levels in 143B cells.

Consistent observations indicated that the mitochondria in 143B exhibited reduced size and increased membrane density, however, the mitochondrial morphology remained normal in the co-culture groups (Figure 7B). To further validate these morphological alterations, we employed ImageJ software for quantitative analysis of mitochondrial area and number. As illustrated in Supplementary Figure S5A, the mitochondrial area and length in untreated 143B cells were markedly decreased (*P* < 0.001), and the number of mitochondria per cell was also significantly reduced (Supplementary Figure S5B, *P* < 0.001). In contrast, cells co-cultured with CAFs or treated with CAFs-derived exosomes exhibited larger and elongated mitochondria, with preserved mitochondrial density and cristae structure. These findings indicated that exosomal COX4I2 contributed to the maintenance of mitochondrial integrity through the inhibition of ferroptosis.

The Edu assay results corroborated this trend, demonstrating that the proliferative capacities of 143B co-cultured with CAFs and CAFs derived exosomes were markedly enhanced compared to 143B cells (Figure 7C, *P* < 0.001).

Additionally, the ROS measurement results were consistent with these findings, showing that the ROS signal intensity was weaker in 143B co-cultured with CAFs and CAFs derived exosomes compared to 143B (Figure 7D).

To further validate the role of exosomal COX4I2, we upregulated the expression of the COX4I2 protein in CAFs using plasmid transfection, extracted the corresponding exosomes, and subsequently co-cultured with 143B for 24 h.

**FIGURE 8:**
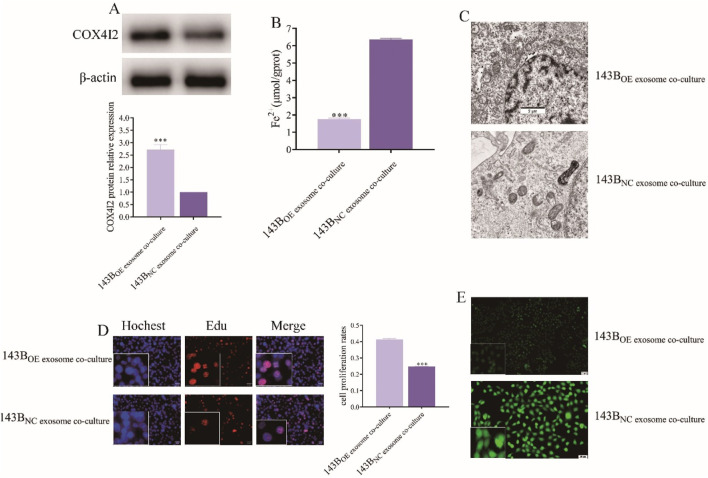
The proliferation of 143B was facilitated by the inhibition of ferroptosis through the overexpression of COX4I2 protein in exosomes derived from CAFs. **(A)** Western blot analysis of COX4I2 protein expression in 143B following co-culture with CAFs exosomes in different treatments. **(B)** Images of Fe^2+^ accumulation detection in 143B in different treatment groups. **(C)** Transmission electron microscopy images of mitochondrial morphology in 143B in different treatment groups. **(D)** Fluorescence-based Edu assay results of 143B in different treatment groups. **(E)** Fluorescence detection results of ROS levels in 143B in different treatment groups. Statistical analysis: One-way ANOVA; n = 3 replicates. Data presented as mean ± SD. ***P < 0.001.

Compared to 143B co-cultured with CAFs exosomes transfected with NC plasmids, the expression of the COX4I2 protein was significantly increased in 143B co-cultured with CAFs exosomes transfected with OE plasmids (Figure 8A) (*P* < 0.001). The Fe^2+^ detection results showed that 143B co-cultured with CAFs exosomes transfected with NC plasmids exhibited higher Fe^2+^ accumulation, whereas those co-cultured with exosomes transfected with OE plasmids showed reduced Fe^2+^ accumulation (Figure 8B, *P* < 0.001). Furthermore, MDA (Supplementary Figure S9B, *P* < 0.001) and ACSL4 (Supplementary Figure S10B, *P* < 0.001) detection demonstrated that 143B cells co-cultured with exosomes derived from CAFs transfected with the NC plasmid exhibited elevated MDA levels, whereas those co-cultured with exosomes from CAFs transfected with the OE plasmid displayed reduced MDA levels.

Similarly, compared to NC group, no significant reduction in mitochondrial membrane density was observed in 143B co-cultured with CAFs exosomes transfected with OE plasmids (Figure 8C).

The cell proliferation assay also revealed that, compared to NC group, the fluorescence intensity of 143B co-cultured with CAFs exosomes transfected with OE plasmids was enhanced, indicating improved cell viability (Figure 8D, *P* < 0.001).

Furthermore, ROS fluorescence detection results indicated that the strong ROS fluorescence signal observed in NC group was significantly diminished in 143B co-cultured with CAFs exosomes transfected with OE plasmids (Figure 8E; Supplementary Figure S8, *P* < 0.001), thereby confirming that the COX4I2 protein derived from CAFs exosomes promoted the malignant proliferation of osteosarcoma cells by inhibiting ferroptosis.

### Exosomal COX4I2 protein plays an oncogene role in osteosarcoma by suppressing ferroptosis *in vivo*

**FIGURE 9:**
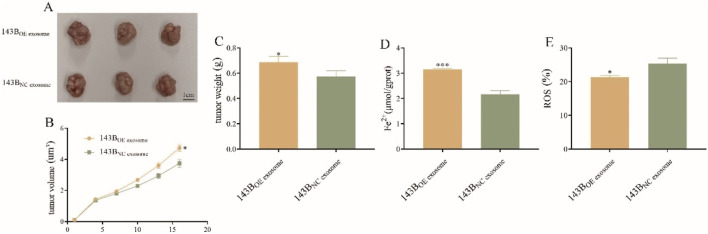
The oncogene role of exosomal COX4I2 protein in osteosarcoma *in vivo* (n = 3/group). **(A)** The tumor, **(B)** tumor volume, and **(C)** tumor weight of exosomal COX4I2 protein group and NC group. **(D)** The Fe^2+^ level and **(E)** ROS levels of exosomal COX4I2 protein group and NC group. Treatment duration: 16 days after tumor establishment. Statistical analysis: Student’s t-test. Data shown as mean ± SD. *P < 0.05, ***P < 0.001.

To further validate the *in vitro* findings, the nude mice were injected with plasmids overexpressing COX4I2 protein and NC, respectively. As illustrated in Figure 9A, tumors in the exosome-treated group were visibly larger than those in the control group. Consistently, tumor volume was significantly greater in the exosome-treated group across the entire treatment period (Figure 9B, *P* = 0.0014), and the final tumor weight was also notably increased (Figure 9C, *P* = 0.0425), indicating that CAF-derived exosomes promote osteosarcoma growth *in vivo*.

IHC staining confirmed that the COX4I2 protein was significantly upregulated in tumor tissues from the overexpression group compared to those in the control group (Supplementary Figure S6A). Additionally, GPX4 expression was markedly elevated in tumors derived from mice treated with overexpression exosome compared to those in the control group (Supplementary Figure S6B).

The results of Fe^2+^ detection and ROS level detection simultaneously demonstrated that, compared with NC group, mice overexpressing COX4I2 protein exhibited reduced Fe^2+^ accumulation (Figure 9D, *P* < 0.001) and ROS production (Figure 9E, *P* = 0.015) in the tissues.

Together, these findings were consistent with the results of experiments *in vitro*, demonstrating compelling evidence for exosomal COX4I2 protein’s role in promoting osteosarcoma malignant proliferation through ferroptosis inhibition.

## Discussion

Osteosarcoma is a highly aggressive bone malignancy that predominantly affects pediatric and adolescent populations (Meltzer and Helman, 2021).The biological heterogeneity of osteosarcoma highlights the critical need to identify novel prognostic biomarkers and therapeutic targets. Recent studies have focused on ferroptosis which is a potential vulnerability in cancer therapy (Lei et al., 2024; Hassannia et al., 2019). However, the regulatory mechanisms of ferroptosis in osteosarcoma, especially its modulation by the tumor microenvironment, remain largely uncharacterized.

Firstly, we developed and validated a 12-gene ferroptosis-based prognostic signature using LASSO regression across two independent cohorts. By employing multiple machine learning algorithms and Single-cell RNA analysis, COX4I2 was identified as an oncogene which was enriched in CAFs. Mechanistic investigations demonstrated that the COX4I2 protein was transferred from CAFs to osteosarcoma cells via exosomes, leading to reduced Fe^2+^, ROS accumulation, MDA level, and preserved mitochondrial morphology, and enhanced tumor cell proliferation. These findings were corroborated *in vivo*, where overexpression of exosomal COX4I2 promoted tumor growth and suppressed ferroptosis. Collectively, these results elucidate a novel CAFs exosomal COX4I2/ferroptosis axis that contributed to osteosarcoma progression, providing valuable insights into stromal regulation and potential therapeutic targets.

Several studies have identified prognostic signatures in various tumors, including hepatocellular carcinoma (Bu et al., 2022), lung cancer (Cai et al., 2024), osteosarcoma (Lei et al., 2021), and head and neck squamous cell carcinoma (Chung et al., 2023), based on ferroptosis-related genes. However, these models frequently rely on extensive gene sets without distinguishing between driver genes and non-driver genes, which may compromise their biological specificity. In contrast, this study developed a ferroptosis-related prognostic signature composed solely of driver genes using the TARGET osteosarcoma cohort and further validated its performance in two external GEO datasets. The model exhibited robust predictive capability, effectively stratifying patient survival and demonstrating a strong association with variations in the immune landscape. These findings underscore the clinical significance of ferroptosis as both a prognostic biomarker system and a therapeutic target in osteosarcoma.

Additionally, we employed machine learning algorithms to conduct hub gene screening. The utilization of machine learning represents an emerging trend in biomedical research. Based on the efficient processing of large volumes of genetic data, machine learning can be leveraged to delve deep into gene interactions and unveil their underlying biological mechanisms (Goecks et al., 2020; Perakakis et al., 2018). Forevermore, we employed four different machine learning algorithms to screen hub genes, successfully identifying genes associated with the malignant characterization of osteosarcoma. Notably, all these genes were included in the model construction, further substantiating its robust validity.

To elucidate the cellular context and spatial dynamics of ferroptosis regulators in osteosarcoma, we employed single-cell RNA sequencing analysis. This methodology facilitated the identification of COX4I2 as a hub gene highly enriched in CAFs rather than in tumor cells. Recent evidence underscores the growing significance of CAFs derived exosomes in modulating the tumor microenvironment. In our investigation, we established that COX4I2 was actively transferred from CAFs to osteosarcoma cells via exosomes, reducing intracellular Fe^2+^ and ROS levels, thereby suppressing ferroptosis, and promoting tumor proliferation. Recent studies have demonstrated that exosomal protein cargo can directly regulate oxidative stress in recipient cells. For instance, exosomal peroxiredoxin-1 (PRDX1) enhanced antioxidant defenses and promoted cell survival under conditions of oxidative stress (Mullen et al., 2015). In contrast, exosomal superoxide dismutase-3 (SOD3) imparted resistance to lipid peroxidation in colorectal carcinoma cells (Cao et al., 2024). Collectively, these findings highlight an emerging paradigm in which exosomal proteins, in addition to RNAs, play a crucial role in modulating redox homeostasis within the tumor microenvironment.

COX4I2 (cytochrome c oxidase subunit 4 isoform 2) is a mitochondrial protein that plays a critical role in cellular respiration and oxidative metabolism (Hüttemann et al., 2001; Shteyer et al., 2009). It has been shown to contribute to hypoxic adaptation in various solid tumors by facilitating metabolic reprogramming, angiogenesis, and therapeutic resistance (Pajuelo Reguera et al., 2020; Haghjoo et al., 2020). We provided evidence that COX4I2 derived from CAFs exosomes functioned as an onco-protein, inhibiting ferroptosis in osteosarcoma cells and promoting tumor proliferation both *in vitro* and *in vivo*. This represents the first report identifying COX4I2 as a key regulator of stromal-mediated ferroptosis evasion in osteosarcoma, thereby enhancing our understanding of mitochondrial signaling within the tumor microenvironment. Clinically, these findings have dual implications: firstly, COX4I2 may serve as a prognostic biomarker for patients with bone tumors; secondly, targeting COX4I2 or its exosomal packaging and delivery pathways could potentially enhance the efficacy of ferroptosis-based therapies. Collectively, these results underscore the significance of COX4I2-mediated suppression of ferroptosis in osteosarcoma progression and suggest that disrupting this axis may offer innovative treatment strategies.

In addition to its mechanical function, COX4I2 may serve as a viable therapeutic target. Although no direct inhibitors of COX4I2 are currently available, its transcriptional regulation by hypoxia-inducible factor-1α (HIF-1α) suggests the potential for targeting upstream regulatory pathways (Douiev et al., 2021; Li X. et al., 2024). Furthermore, our findings demonstrated that the inhibition of exosome release, through GW4869, could effectively counteract the tumor-promoting effects mediated by COX4I2 transfer. This indicated that modulating exosome generation was a feasible therapeutic intervention. Collectively, these findings implied that interfering with the communication between stromal and tumor-derived exosomes may represent a promising strategy to enhance sensitivity to ferroptosis and suppress osteosarcoma progression.

Nevertheless, several limitations merit further consideration. First, although the transfer of COX4I2 via CAFs exosomes has been demonstrated, direct labeling and tracking *in vivo* of exosomal COX4I2 would provide stronger causal evidence. Second, our work relied on cell lines and xenografts without validation in patient specimens. Analysis of CAF and exosomal COX4I2 in clinical osteosarcoma samples would be critical for translational relevance. Last but not least, although six candidate genes were identified by machine learning, only COX4I2 was experimentally validated based on its strong cross-algorithm importance, CAF-specific expression, and feasibility for exosomal mechanism investigation. Future studies will explore the biological roles of the remaining genes to further refine the model.

## Conclusion

In conclusion, this study demonstrates that CAFs exosomal COX4I2 serves as a pivotal regulator of osteosarcoma progression by inhibiting ferroptosis. Through the identification of a novel stromal–tumor communication axis, we offer profound insights into the non-cell-autonomous regulation of ferroptosis and highlight COX4I2 as a potential prognostic biomarker and therapeutic target. These results lay the foundation for the development of ferroptosis-sensitizing strategies in osteosarcoma, which may ultimately enhance clinical outcomes in this aggressive malignancy.

## Data Availability

The original contributions presented in the study are included in the article/Supplementary Material, further inquiries can be directed to the corresponding author.
