# Supplementary material for: Multi-transcriptomics analysis of ferroptosis related genes reveals CAFs exosomal COX4I2 as a novel therapeutic target in osteosarcoma
Source: Front Cell Dev Biol. 2025 Sep 4;13:1620648. doi: 10.3389/fcell.2025.1620648 (PMC12443774; doi:10.3389/fcell.2025.1620648)
Supplement: Supplementary file 1 [file DataSheet1.docx]

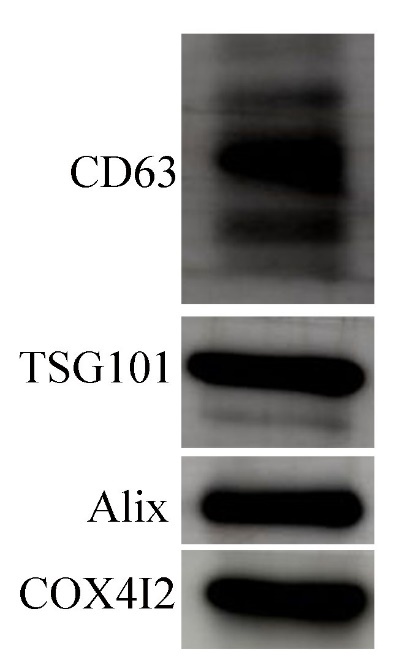
Supplementary Figure

S1 Identification of exosomal markers and COX4I2 in CAF-derived exosomes. n = 3 biological replicates.


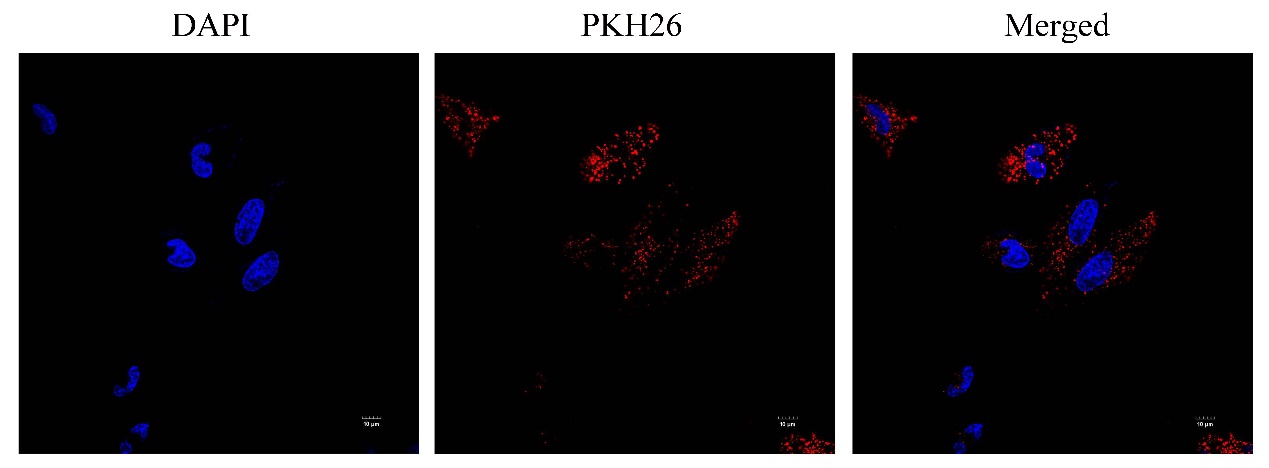


S2 Confocal microscopy results after co-culturing CAFs-derived exosomes with 143B for 24 hours.


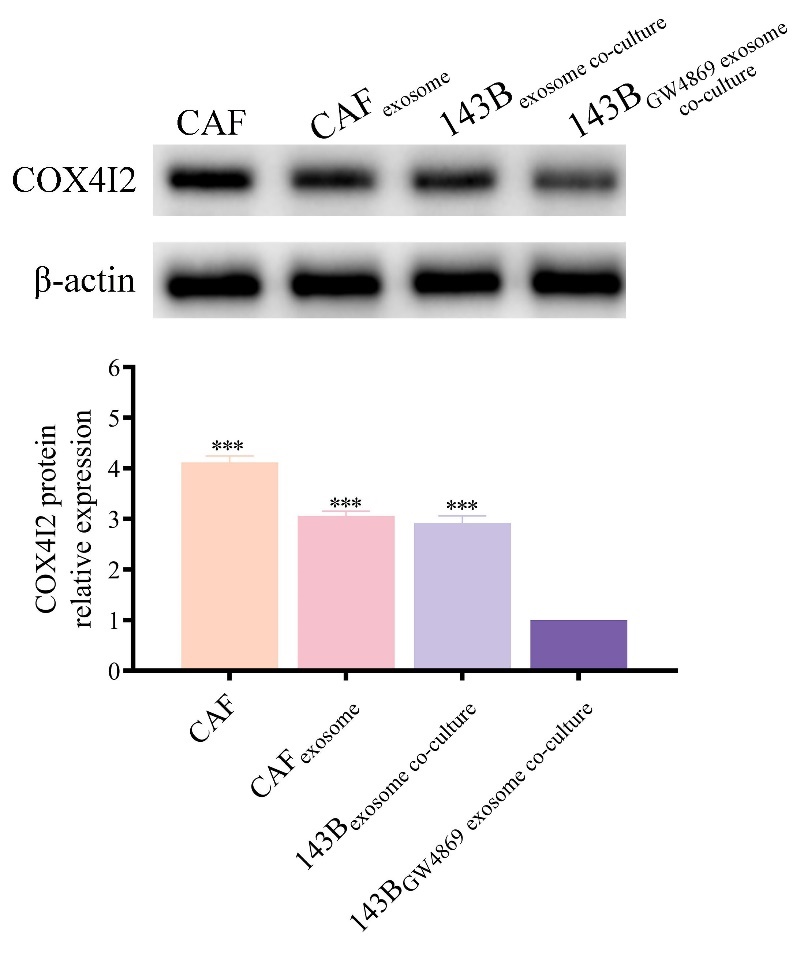


S3 Western blot results of COX4I2 protein expression bands in CAF, CAF_exosome_, 143B_exosome co-culture_, and 143B_GW4869 exosome co-culture_ group. n = 3 biological replicates.


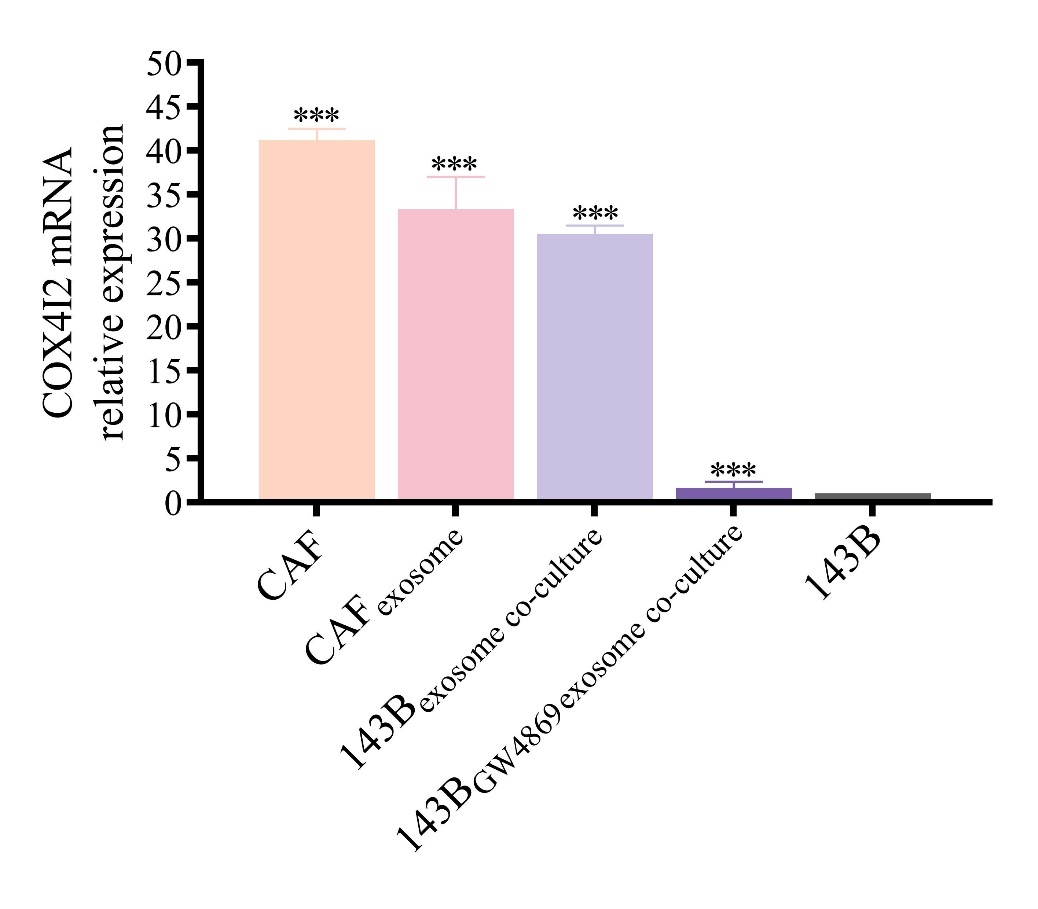


S4 qRT-PCR analysis of COX4I2 mRNA expression in CAF, CAF_exosome_, 143B _exosome co-culture,_ 143B_GW4869 exosome co-culture_, and 143B group. n = 3 biological replicates.


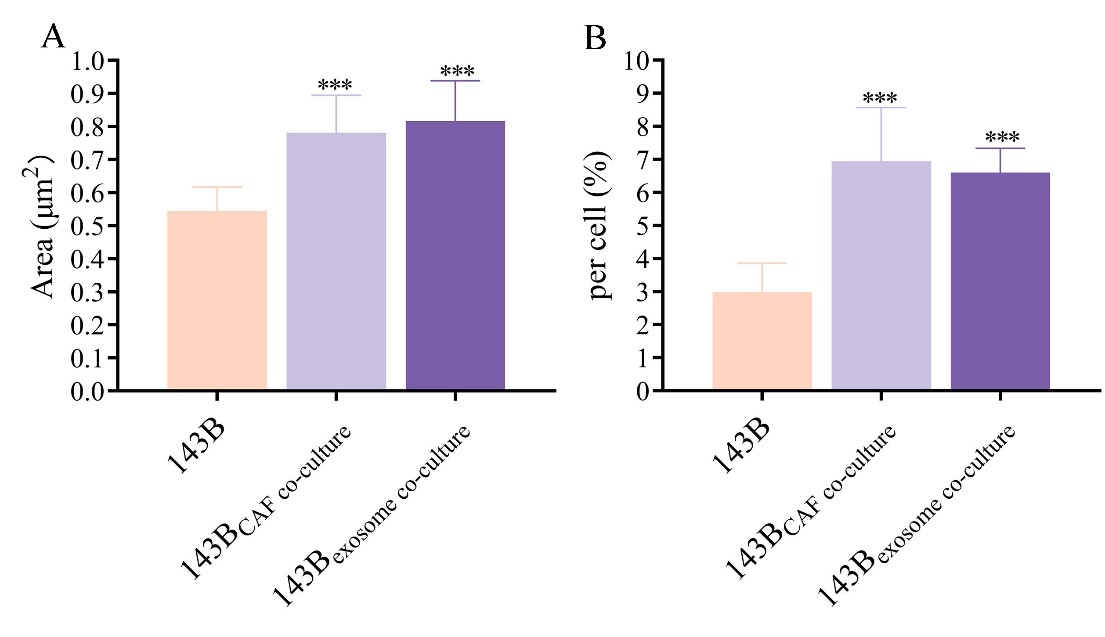


S5 Quantitative analysis of mitochondrial morphology in 143B cells by TEM. (A) Mitochondrial area (μm²) in CAFs- and exosome-treated groups. (B) The number of mitochondria per cell in CAFs/exosome groups. n = 3 biological replicates.


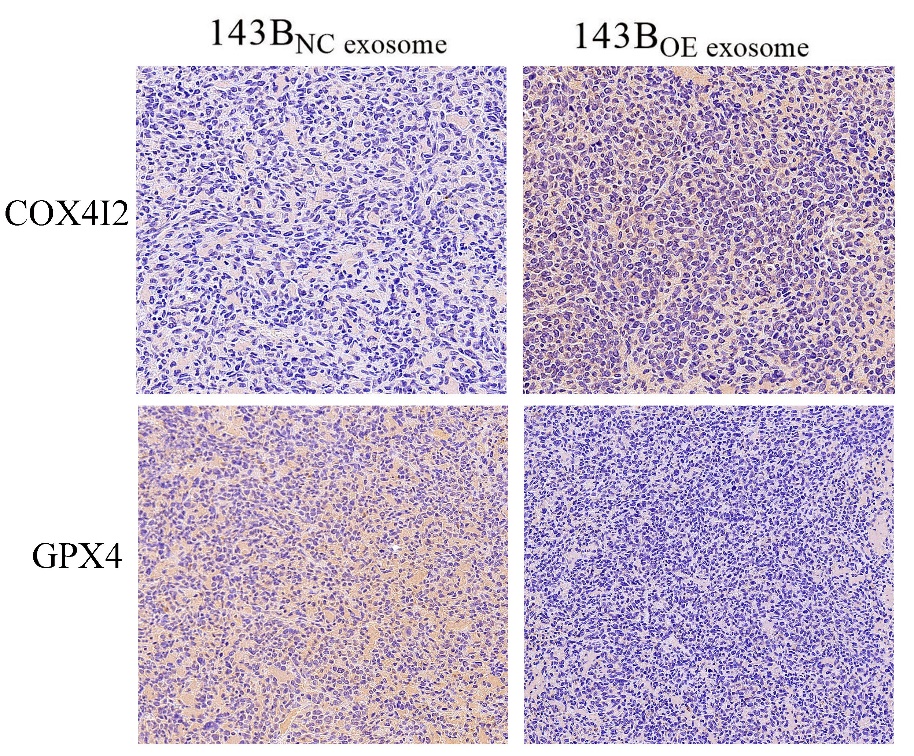


S6 IHC staining of COX4I2 and GPX4 in xenograft tumor tissues. n = 3 biological replicates.

| Supplementary Table S1 List of 68 ferroptosis driver genes | | |
| --- | --- | --- |
| No. | Gene Symbol | FerrDb Category |
| 1 | ACSL1 | Driver |
| 2 | ALOX12 | Driver |
| 3 | ALOX15 | Driver |
| 4 | ALOX15B | Driver |
| 5 | ALOX5 | Driver |
| 6 | ALOXE3 | Driver |
| 7 | ATG7 | Driver |
| 8 | CD82 | Driver |
| 9 | COX4I2 | Driver |
| 10 | CHAC1 | Driver |
| 11 | CS | Driver |
| 12 | CTSB | Driver |
| 13 | CYB5R1 | Driver |
| 14 | CYGB | Driver |
| 15 | DDR2 | Driver |
| 16 | DLD | Driver |
| 17 | DPP4 | Driver |
| 18 | ELAVL1 | Driver |
| 19 | ELOVL5 | Driver |
| 20 | EMC2 | Driver |
| 21 | EPAS1 | Driver |
| 22 | FADS1 | Driver |
| 23 | FADS2 | Driver |
| 24 | FAR1 | Driver |
| 25 | FLT3 | Driver |
| 26 | HILPDA | Driver |
| 27 | FTH1 | Driver |
| 28 | G6PD | Driver |
| 29 | GJA1 | Driver |
| 30 | GLS2 | Driver |
| 31 | LIFR | Driver |
| 32 | GSK3B | Driver |
| 33 | GSTZ1 | Driver |
| 34 | H19 | Driver |
| 35 | HIlpda | Driver |
| 36 | KEAP1 | Driver |
| 37 | MAP3K11 | Driver |
| 38 | MAPK9 | Driver |
| 39 | MBOAT1 | Driver |
| 40 | PRARG | Driver |
| 41 | PRKCA | Driver |
| 42 | PRKAA1 | Driver |
| 43 | METTL14 | Driver |
| 44 | LONP1 | Driver |
| 45 | MTDH | Driver |
| 46 | MTOR | Driver |
| 47 | SLC25A28 | Driver |
| 48 | SLC38A1 | Driver |
| 49 | PRMT3 | Driver |
| 50 | PRNP | Driver |
| 51 | SLC39A14 | Driver |
| 52 | SLC39A7 | Driver |
| 53 | SNCA | Driver |
| 54 | RPL8 | Driver |
| 55 | SAT1 | Driver |
| 56 | SNX5 | Driver |
| 57 | SOCS1 | Driver |
| 58 | SLC1A5 | Driver |
| 59 | SLC7A11 | Driver |
| 60 | TGFBR1 | Driver |
| 61 | TIMP1 | Driver |
| 62 | TLR4 | Driver |
| 63 | TRIM21 | Driver |
| 64 | TRIM46 | Driver |
| 65 | TFRC | Driver |
| 66 | TP53 | Driver |
| 67 | VDAC2 | Driver |
| 68 | YAP1 | Driver |


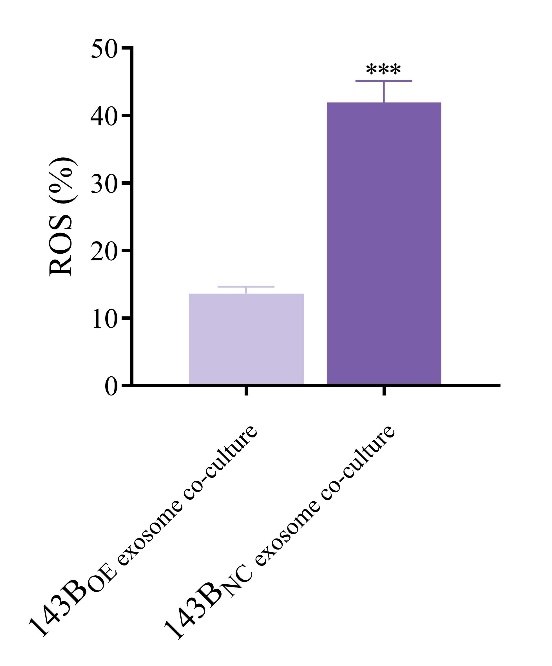


S7 Quantification of ROS fluorescence intensity in 143B cells co-cultured with CAFs exosomes in different treatments.

| Supplementary Table S2. Multivariate Cox Regression Analysis of Risk Score and Clinical Characteristics | | | |
| --- | --- | --- | --- |
| Variable | HR | 95% CI | *P* value |
| Age | 1.052 | 0.496, 1.993 | 0.764 |
| Gender | 2.613 | 0.881, 3.659 | 0.913 |
| Clinical stage | 0.597 | 0.169, 1.052 | 0.094 |
| Metastasis | 1.443 | 0.932, 2.436 | 0.528 |
| Risk_Score | 3.618 | 3.066, 4.192 | **<0.001** |


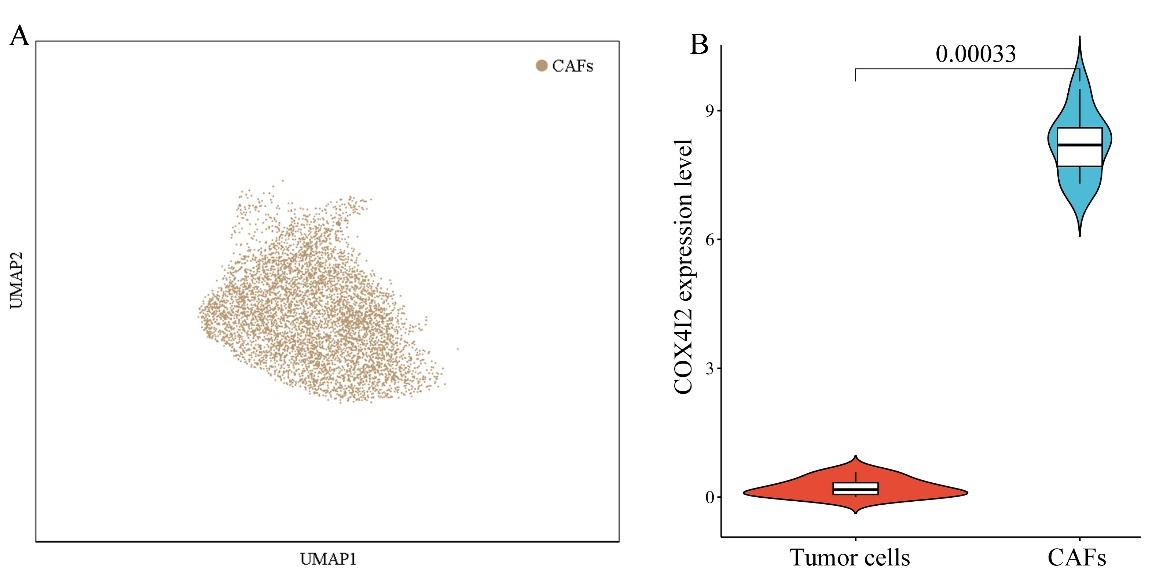


S8 Single-cell RNA-seq analysis reveals CAF-specific enrichment of COX4I2 expression. (A) UMAP plotof the clustering of osteosarcoma cell populations with COX4I2 expression overlay. (B) Violin plot of COX4I2 expression levels across tumor cells and CAFs.


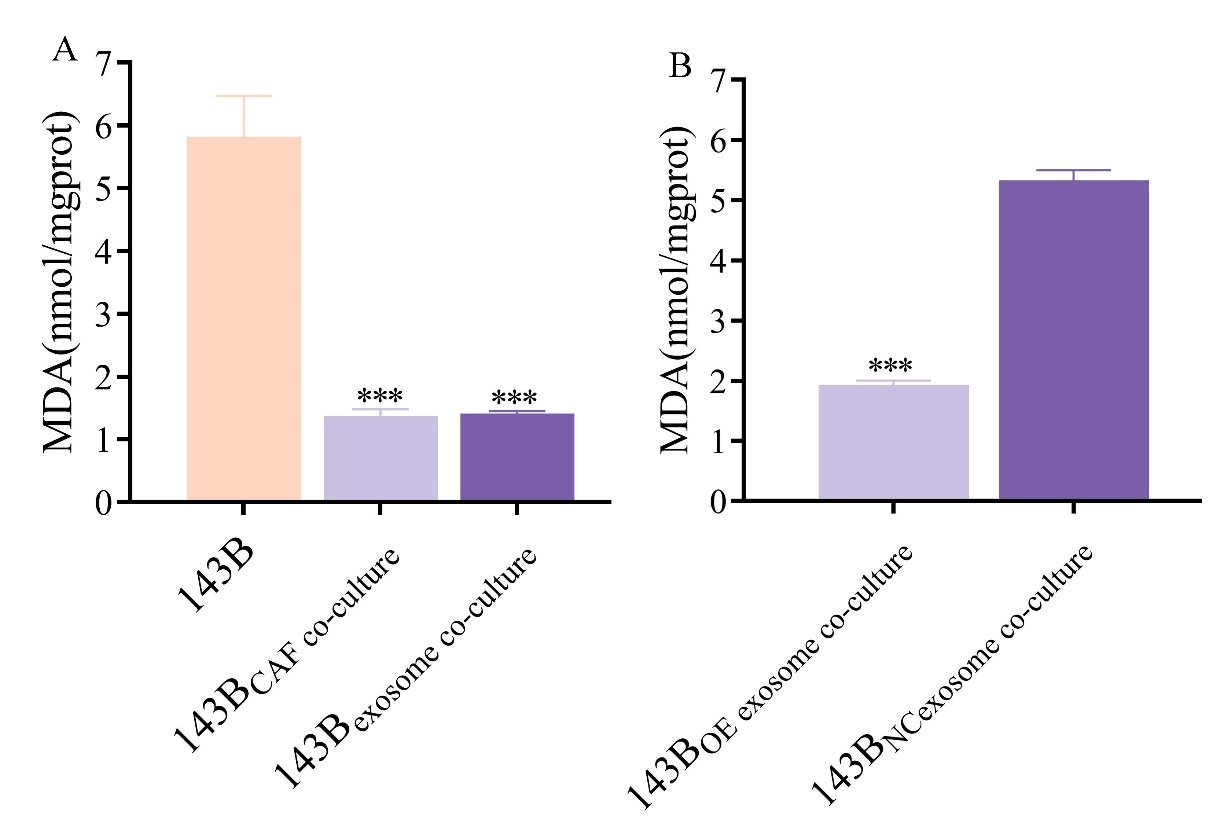


S9 MDA assay in 143B cells treated with CAF cells, CAFs-derived exosomes (A), COX4I2 OE exosome, and NC exosome (B), respectively.


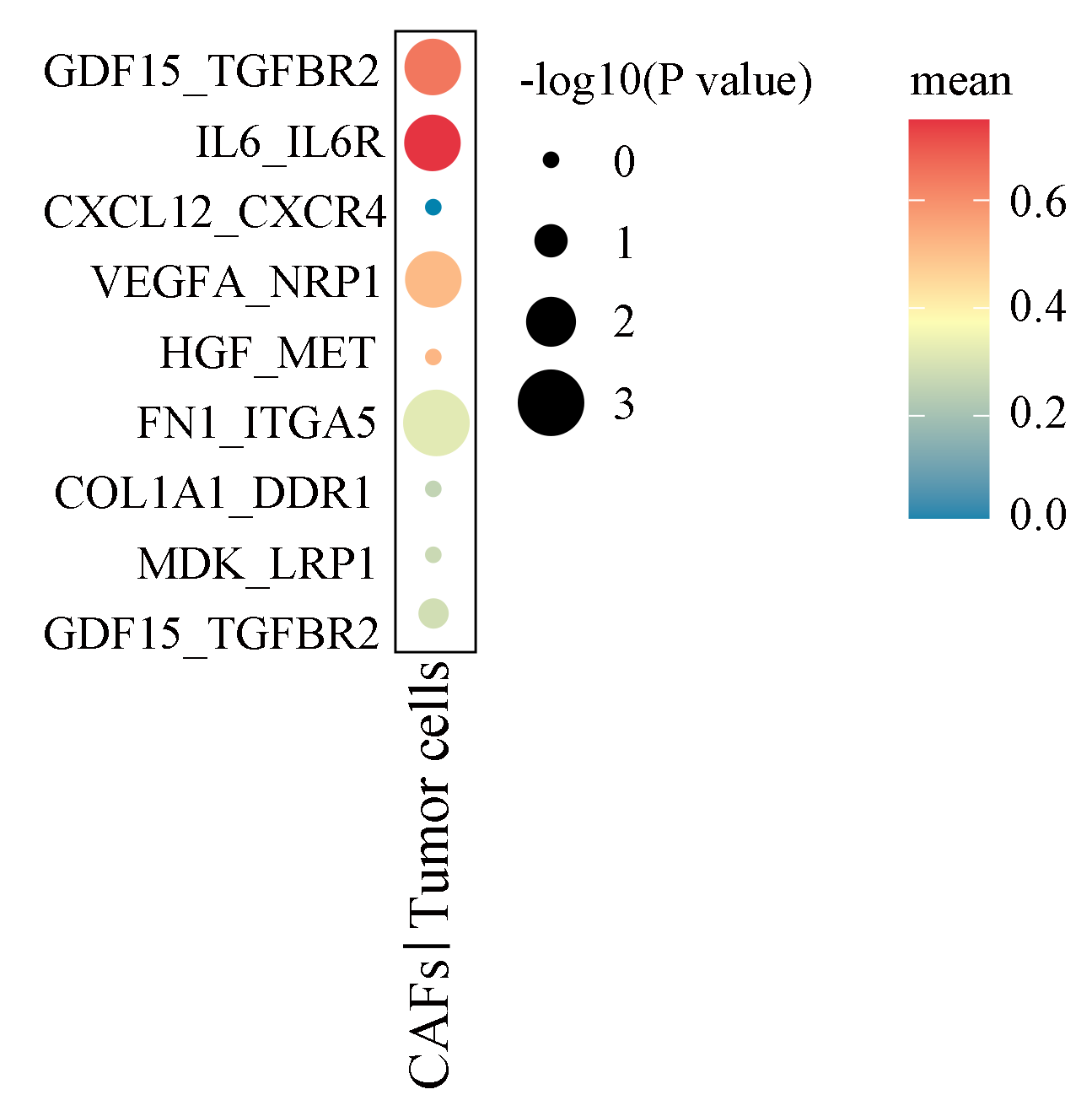


S10 Predicted ligand – receptor interactions between CAFs and osteosarcoma cells based on single-cell transcriptomic analysis.


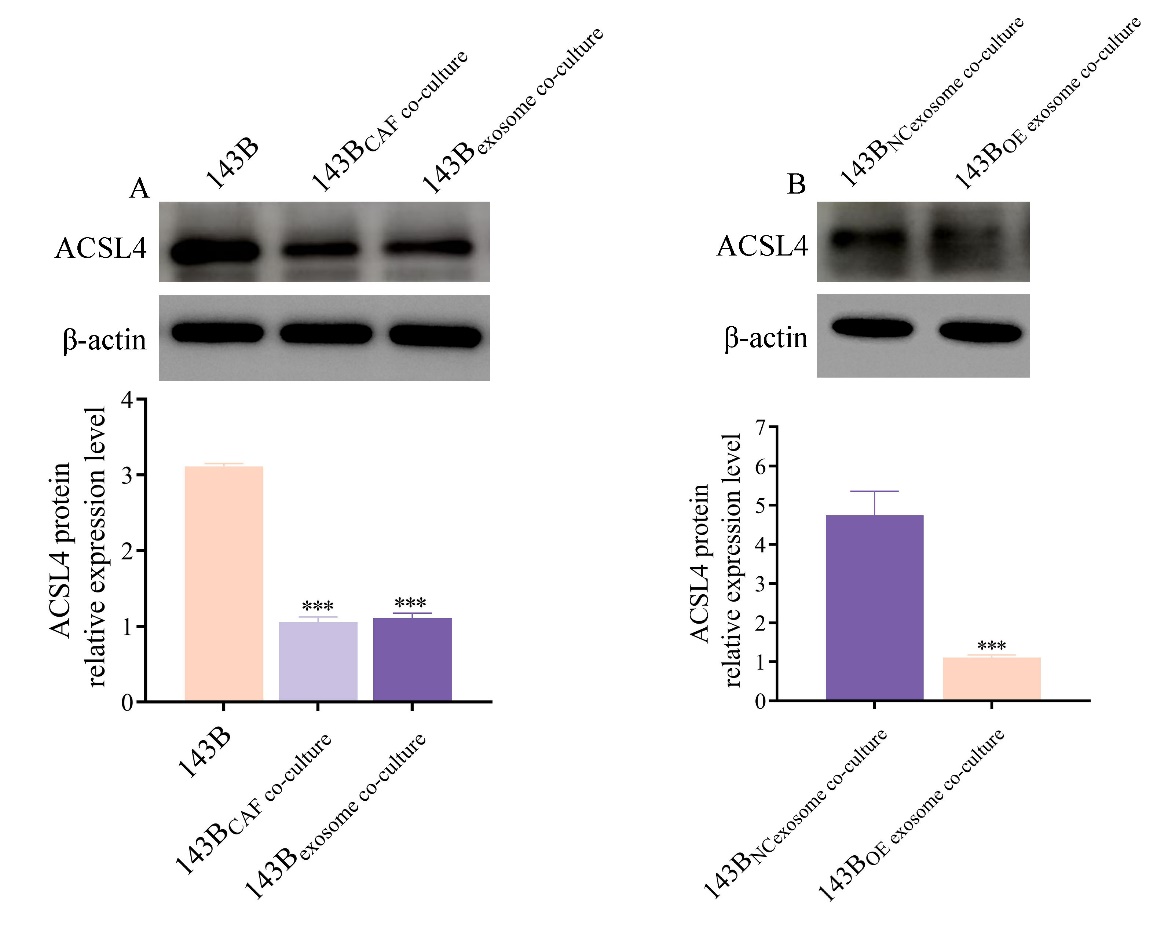


S11 Western blot results of ACSL4 protein expression in 143B cells co-cultured with CAFs and CAFs exosomes (A), and OE exosome, and NC exosome (B), respectively.
